# RosE represses Std fimbrial expression in *Salmonella enterica* serotype Typhimurium

**DOI:** 10.1111/j.1365-2958.2008.06185.x

**Published:** 2008-05-01

**Authors:** Daniela Chessa, Maria G Winter, Sean-Paul Nuccio, Çagla Tükel, Andreas J Bäumler

**Affiliations:** Department of Medical Microbiology and Immunology, School of Medicine, University of California at Davis One Shields Ave., Davis, CA 95616-8645, USA

## Abstract

The *Salmonella enterica* serotype Typhimurium (*S. typhimurium*) genome contains a large repertoire of putative fimbrial operons that remain poorly characterized because they are not expressed *in vitro*. In this study, insertions that induced expression of the putative *stdABCD* fimbrial operon were identified from a random bank of transposon mutants by screening with immuno-magnetic particles for ligand expression (SIMPLE). Transposon insertions upstream of *csgC* and *lrhA* or within *dam*, *setB* and *STM4463* (renamed *rosE*) resulted in expression of StdA and its assembly into fimbrial filaments on the cell surface. RosE is a novel negative regulator of Std fimbrial expression as indicated by its repression of a *std*::*lacZ* reporter construct and by binding of the purified protein to a DNA region upstream of the *stdA* start codon. Expression of Std fimbriae in the *rosE* mutant resulted in increased attachment of *S. typhimurium* to human colonic epithelial cell lines (T-84 and CaCo-2). A *rosE* mutant exhibited a reduced ability to compete with virulent *S. typhimurium* for colonization of murine organs, while no defect was observed when both competing strains carried a *stdAB* deletion. These data suggest that a tight control of Std fimbrial expression mediated by RosE is required during host pathogen interaction.

## Introduction

Laboratory-grown cultures of *Salmonella enterica* serotype Typhimurium (*S. typhimurium*) commonly elaborate only type 1 fimbriae (Duguid *et al*., 1966) encoded by the *fim* operon (Clegg *et al*., 1987) and thin curled fimbriae (also known as thin aggregative fimbriae or curli) (Grund and Weber, 1988; Stolpe *et al*., 1994) encoded by the *csg* (*agf*) gene cluster (Romling *et al*., 1998). Two other *S. typhimurium* fimbrial gene clusters, termed *pef* (Friedrich *et al*., 1993) and *lpf* (Bäumler and Heffron, 1995), induce expression of fimbriae when the cloned genes are introduced into *Escherichia coli*. However, nucleotide sequencing identified nine additional putative fimbrial operons in the *S. typhimurium* genome, termed *bcf* (Tsolis *et al*., 1999), *stf* (Emmerth *et al*., 1999; Morrow *et al*., 1999), *saf* (Folkesson *et al*., 1999), *stb*, *stc*, *std*, *sth*, *sti* and *stj* (McClelland *et al*., 2001). The fact that nine putative fimbrial operons were only identified by sequence analysis in this genetically well-characterized organism reflects the tight control of their expression *in vitro*, which is detectable neither by Western blot (Humphries *et al*., 2005) nor by flow cytometry (Humphries *et al*., 2003) after growth of *S. typhimurium* under standard laboratory conditions. This lack of *in vitro* expression has prevented a thorough functional characterization of the encoded adhesins.

The *std* operon was initially identified during sequence analysis of the human-adapted *S. enterica* serotype Typhi strain CT18 (Townsend *et al*., 2001). Many of the putative fimbrial operons identified in the genomes of human-adapted *Salmonella* serotypes, including *S. enterica* serotypes Paratyphi A and Typhi (strains CT18 and Ty2), contain pseudogenes (i.e. genes carrying frameshift mutations or stop codons) (Parkhill *et al*., 2001; Deng *et al*., 2003; McClelland *et al*., 2004). Interestingly, only two fimbrial operons, *tcf* and *std*, share the following characteristics: they are present and apparently intact in all three available genomes of human-adapted *Salmonella* serotypes (Andrews-Polymenis and Bäumler, 2006). DNA–DNA hybridization studies show that the majority of *Salmonella* serotypes investigated contain orthologues of the *std* operon (Townsend *et al*., 2001; Porwollik *et al*., 2002; 2004; Chan *et al*., 2003; Anjum *et al*., 2005). Sequence comparison of its usher protein identifies the *std* operon as a member of the π-fimbriae, a group including well-characterized virulence factors such as pyelonephritis-associated (P) fimbriae of *E. coli* and the Mannose-resistant/*Proteus*-like (MR/P) fimbriae of *Proteus mirabilis* (Nuccio and Bäumler, 2007). Deletion of the *std* operon in *S. typhimurium* causes a competitive disadvantage during intestinal persistence in a mouse model (Weening *et al*., 2005). Mice infected with *S. typhimurium* seroconvert to StdA, which provides indirect evidence for *in vivo* expression of the *std* operon (Humphries *et al*., 2005). Gene expression profiling of an *S. typhimurium dam* mutant recently demonstrated that transcription of the *std* operon is repressed by Dam methylation (Balbontin *et al*., 2006). However, as the elaboration of surface structures has not been demonstrated in *S. typhimurium*, *std* must still be considered a putative fimbrial operon.

In this study we used a genetic screen to identify novel genes silencing expression of the *std* operon *in vitro* and demonstrated for the first time expression of the encoded fimbriae in *S. typhimurium*. Our results represent an important first step in characterizing Std fimbriae and lead the way for future functional studies on the remaining putative fimbrial operons present in the *S. typhimurium* genome.

## Results

### Identification of *S. tyhimurium* mutants expressing StdA using immuno-magnetic particles for ligand expression (SIMPLE)

To identify novel regulatory genes controlling expression of StdA *in vitro*, we generated mutant libraries in an *S. typhimurium fim* mutant (AJB4) using the transposons Mu*d*-Cam (Elliott, 1993) and T-POP (Rappleye and Roth, 1997). Approximately 18 500 Mu*d*-Cam mutants were generated, approximately 40% of which resulted from homologous recombination of the transposon into the *hisD* gene (data not shown), because the transposon was transduced from a donor strain (TE3461) carrying a *hisD*::Mu*d*-Cam insertion. Thus, the Mu*d*-Cam mutant bank contained an estimated 11 000 random insertion mutants. In addition, a bank containing approximately 9000 random T-POP insertion mutants was generated in an *S. typhimurium fim* mutant (AJB4). To this end, a plasmid encoding a Tn*10* transposase with broad target specificity (pNK2880) was introduced into the *S. typhimurium fim* mutant. The T-POP transposon was delivered into this strain by transduction from an *S. typhimurium* donor (TH3923) (Lee *et al*., 2007) carrying the transposon on an *E. coli* F′ plasmid. A derivative of *S. typhimurium* isolate SR11 was used for this procedure, because this strain can be transduced with P22 to chloramphenicol (Mu*d*-Cam) or tetracycline (T-POP) resistance, but is resistant to P22-mediated lysis, thus avoiding a selection for phage-resistant mutants during culture of mutant pools.

**Fig. 1:**
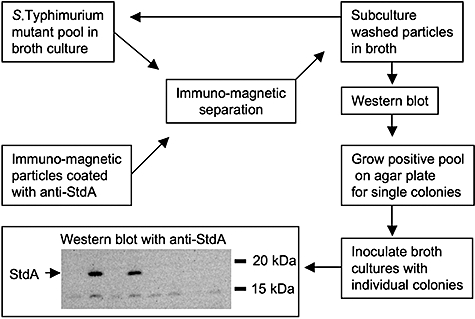
Flow chart of the SIMPLE approach used to identify *S. typhimurium* mutants expressing StdA. The bottom panel shows a Western blot detected with rabbit anti-StdA serum. Each lane represents a culture inoculated with a single colony from a mutant pool after two rounds of enrichment with immuno-magnetic particles coated with anti-StdA serum. The molecular mass of standard proteins is indicated on the right.

The immuno-magnetic particles for ligand expression (SIMPLE) approach (Nuccio *et al*., 2007) is based on the idea that incubation of a mutant bank with magnetic particles coated with antiserum raised against a fimbrial protein can be used to enrich for fimbriated mutants by immuno-magnetic separation (Nuccio *et al*., 2007). The Mu*d*-Cam and T-POP mutant banks were divided into 16 and 7 pools, respectively, and each pool was used to inoculate a static Luria–Bertani (LB) broth (pH 7) culture with (for T-POP) or without (for Mu*d*-Cam) tetracyline. Each pool was incubated with magnetic particles coated with anti-StdA serum; particle-bound bacteria were re-suspended in broth and used to inoculate a second static broth culture to repeat the enrichment procedure. After two enrichment cycles, a sample from each pool was grown statically in LB broth and expression of StdA by bacteria in the pool was assessed by Western blot (Fig. 1). Four T-POP pools and five Mu*d*-Cam pools were positive for StdA expression, suggesting that enrichment for a mutant expressing this protein had occurred. Twenty bacterial colonies from each positive pool were grown statically in LB broth to identify individual transposon mutants expressing StdA. One StdA-expressing mutant from each pool was selected, a lysate was prepared using phage KB1 *int* (because SR11 derivatives are resistant to P22-mediated lysis) and the transposon insertion was transduced into an *S. typhimurium fim* mutant (AJB4). Transduction yielded four T-POP mutants (CD6R, CD7R, CD12R and CD15R) and five Mu*d*-Cam mutants (CD2R, CD8R, CD10R, DB2R and DB3R) that expressed StdA in LB broth.

### Identification of transposon insertion sites

**Fig. 2:**
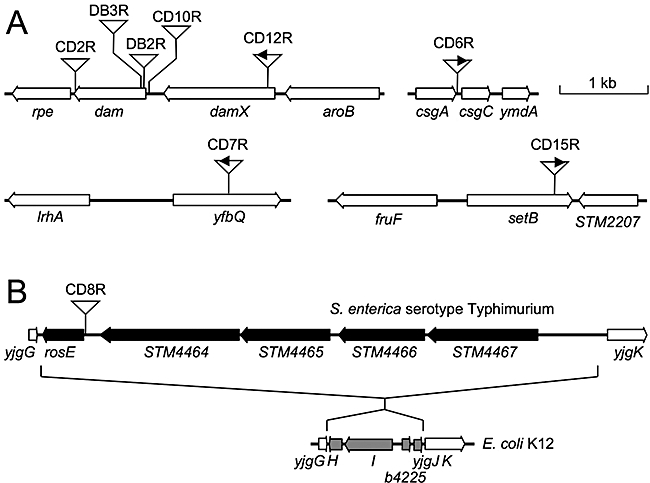
Transposon insertion sites in *S. typhimurium* mutants expressing StdA. A. Arrows indicate the size and orientation of genes in the *S. typhimurium* genome. The location of transposon insertions is indicated above each genetic map. Arrowheads indicate the orientation of the *tet* promoter of T-POP (CD12R, CD6R, CD7R, CD15R). B. Comparison of the *yjgG–yjgK* intergenic regions of *S. typhimurium* strain LT2 (top) and *E. coli* strain K12 (bottom). Closed arrows indicate the size and orientation of genes present in the *S. typhimurium* genome but absent from *E. coli* K12. Open arrows indicate genes present in *S. typhimurium* and *E. coli*. Grey arrows indicate genes present in *E. coli* K12 but absent in *S. typhimurium*.

DNA regions flanking the T-POP transposon insertion sites in strains CD2R, CD6R, CD7R, CD8R, CD12R, CD10R, CD15R, DB2R and DB3R were cloned by inverse polymerase chain reaction (PCR) and the respective nucleotide sequences were determined. Four Mu*d*-Cam insertion sites were either within or immediately upstream of the *dam* open reading frame, at base pair positions +803 (CD2R), +36 (DB3R), +10 (DB2R) or −40 (CD10R) relative to the start codon. One T-POP insertion was located in *damX*, the open reading frame located directly upstream of *dam*, at base pair position +20 (CD12R) relative to the start codon (Fig. 2). The deoxyadenosine methylase (Dam) encoded by the *dam* gene has previously been implicated in controlling expression of fimbriae in *E. coli* (Blyn *et al*., 1990; van der Woude and Low, 1994) and *S. typhimurium* (Nicholson and Low, 2000).

The T-POP insertion in CD6R was located in the *csgA csgC* intergenic region, at base pair position −60 relative to the *csgC* start codon. The *csg* gene cluster is involved in the biosynthesis of thin curled fimbriae in *S. typhimurium* (Romling *et al*., 1998); however, no function in fimbrial biosynthesis has been assigned to the *csgC* open reading frame. One T-POP insertion (CD15R) was found in the *setB* gene, at base pair position +1145 relative to the start codon. The *setB* gene encodes a sugar transporter (Liu *et al*., 1999) and mutations have been associated with pleiotrophic phenotypes, including a delay in chromosome segregation (Espeli *et al*., 2003).

**Fig. 3:**
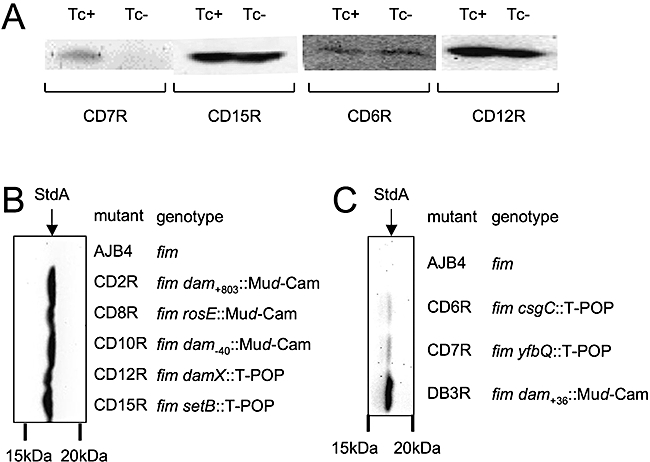
Expression of StdA by *S. typhimurium* mutants. A. Expression of StdA detected by Western blot in T-POP insertion mutants (CD7R, CD15R, CD6R and CD12R) grown in the presence (Tc+) or absence (Tc−) of tetracycline. B. Expression of StdA (arrow) detected after short (20 min) exposure of a Western blot. Bacterial strains analysed are indicated on the right. The molecular mass of standard proteins is indicated at the bottom. C. Expression of StdA (arrow) detected after overnight exposure of a Western blot. Bacterial strains analysed are indicated on the right. The molecular mass of standard proteins is indicated at the bottom.

One T-POP mutant (CD7R) did not express StdA after growth in LB broth in the absence of tetracycline (Fig. 3), suggesting that the *tetA* promoter of T-POP may drive expression of a positive regulatory element in this mutant. The T-POP insertion in CD7R was located in the *yfbQ* open reading frame, at base pair position +600 relative to the start codon. Mutant CD7R carried the T-POP insertion in an orientation in which the *lrhA* gene is located downstream of the *tetA* promoter (Fig. 2). LrhA is a negative regulator of flagellar, chemotactic, motility and type 1 fimbrial gene expression in *E. coli* (Lehnen *et al*., 2002; Blumer *et al*., 2005).

One Mu*d*-Cam insertion was located upstream of open reading frame STM4463 (from hereon referred to as *rosE*), at base pair position −40 relative to the start codon. The protein encoded by *rosE* had homology to transcriptional regulators and showed the highest sequence identity to the ArgR (29%) (Lim *et al*., 1987) and Fur (16%) (Schäffer *et al*., 1985) proteins of *E. coli*. The *rosE* gene was located on an approximately 5 kb islet that was found to be present in all sequenced genomes of *Salmonella* serotypes and in genomes of uropathogenic *E. coli*, but absent from other sequenced *E. coli* genomes (Fig. 2). Open reading frames on this islet encode a putative arginine deiminase system composed of arginine deiminase (STM4467), carbamate kinase (STM4466), ornithine carbamyltransferase (STM4465) and an arginine-ornithine antiporter (STM4464). Similar arginine deiminase operons have been described in both Gram-positive and Gram-negative bacteria where they are involved in catabolizing arginine to ornithine, ammonia and carbon dioxide with the concomitant production of ATP (Luthi *et al*., 1986; 1990; Maghnouj *et al*., 1998).

### StdA expression in *S. typhimurium* mutants is associated with enhanced transcription from the *std* promoter

Analysis of StdA expression divided mutants identified by SIMPLE into those exhibiting a strong signal by Western blot minutes after exposure (Fig. 3B) and those in which a signal was only detectable after prolonged exposure of Western blots (Fig. 3C). Mutants with high levels of StdA expression included *rosE*::Mu*d*-Cam (CD8R), *setB*::Mu*d*-Cam (CD15R), *dam*_+803_::Mu*d*-Cam (CD2R), *dam*_−40_::Mu*d*-Cam (CD10R), *dam*_+10_::Mu*d*-Cam (DB2R) and *damX*::T-POP (CD12R). The *damX*::T-POP mutant (CD12R) expressed StdA when cultures were inoculated from glycerol stocks, but not when cultures were inoculated from a colony grown on LB agar plates, which may result from selection for compensatory mutations (however, this was not further analysed). None of the other mutants exhibited this phenotype. Mutants with lower levels of StdA expression included *yfbQ*::T-POP (CD7R) and *csgC*::T-POP (CD6R), while the *dam*_+36_::Mu*d*-Cam mutant (DB3R) exhibited an intermediate level of expression (Fig. 3C).

**Fig. 4:**
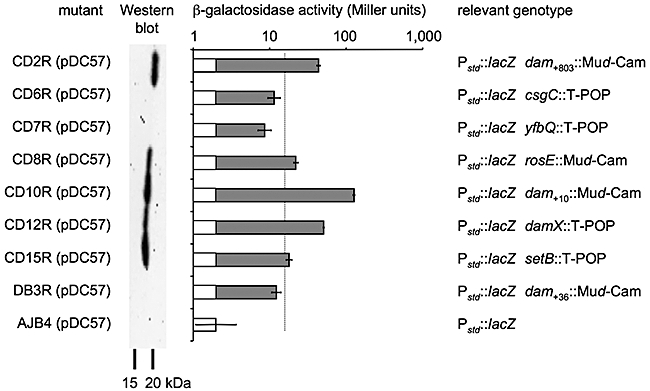
Transcription of the *stdA* gene in *S. typhimurium* strains detected with a plasmid-based (pDC57) *lacZ* transcriptional fusion (P_*std*_::*lacZ*). Bacterial strains analysed are indicated on the right. The second panel from the left shows a Western blot of each strain detected with anti-StdA serum. The molecular mass of standard proteins is indicated at the bottom. The β-galactosidase activities detected in each strain are shown at the centre. Each bar represents the average from three independent experiments ± standard deviation. The genotype of each strain is given on the right.

To determine whether expression of StdA in individual mutants was accompanied with increased expression of the *std* operon, the promoter region upstream of *stdA* (P_*std*_) was introduced into vector pUJ10 oriented such that it controlled expression of a promoterless *lacZ* gene. The resulting plasmid (pDC57) was introduced into each of the mutants and their isogenic parent (AJB4). The parent strain AJB4(pDC57) expressed only low levels of β-galactosidase activity (approximately 2 Miller units on average) while all the T-POP or Mu*d*-Cam insertions characterized in this study exhibited a significantly increased expression of the P_*std*_::*lacZ* reporter construct. The lowest levels of β-galactosidase expression were detected in the *yfbQ*::T-POP mutant (fivefold induction) and the *csgC*::T-POP mutant (sixfold induction) (Fig. 4), which also exhibited the weakest expression of StdA by Western blot (Fig. 3C). A stronger induction of the P_*std*_::*lacZ* reporter construct was detected in the *setB*::Mu*d*-Cam mutant (ninefold induction), the *rosE*::Mu*d*-Cam mutant (11-fold induction), the *damX*::T-POP mutant (26-fold induction) and the *dam* mutants. Expression of StdA was detected after short exposure of Western blots in all mutants in which β-galactosidase expression from the P_*std*_::*lacZ* reporter construct was increased ninefold or higher compared with the parental strain (Fig. 4). These data suggest that expression of StdA in the T-POP or Mu*d*-Cam mutants identified in this study were at least in part due to increased expression from the P_*std*_ promoter. However, expression of the P_*std*_::*lacZ* reporter fusion below the threshold level observed in the *setB*::Mu*d*-Cam mutant resulted in a dramatic reduction of StdA expression detected by Western blot (Fig. 4) to levels only detectable after prolonged exposure (Fig. 3C). Due to the relatively low level of StdA expression detected in the *yfbQ*::T-POP mutant (CD7R) and the *csgC*::T-POP mutant (CD6R) (Fig. 3C), we did not further analyse the respective mutations.

### StdA assembles into fimbrial filaments on the surface of *S. typhimurium* that mediate attachment to human intestinal epithelial cells

**Fig. 5:**
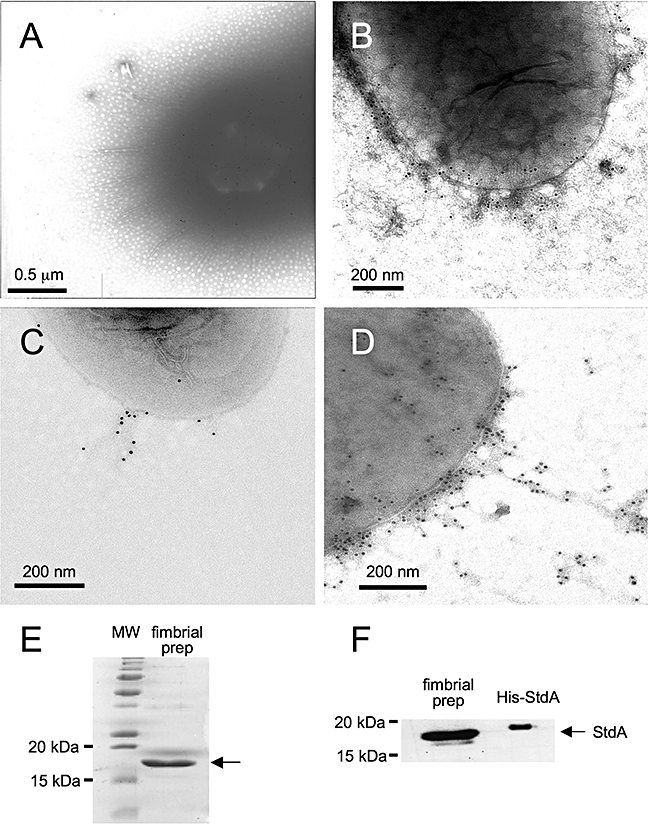
Expression of fimbriae on the surface of *S. typhimurium* transposon mutants investigated by electron microscopy and purification of fimbriae. A. Expression of fimbrial filaments on the surface of a *dam*_+803_::Mu*d*-Cam mutant (CD2R) was visualized by negative staining. B–D. Immunoelectron microscopy detecting expression of StdA on the surface of a *dam*_+803_::Mu*d*-Cam mutant (CD2R) (B), a *rosE*::Mu*d*-Cam mutant (CD8R) (C) and a *setB*::T-POP mutant (CD15R) (D). E and F. Coomassie stain (E) and Western blot (F) of a fimbrial preparation (fimbrial prep) obtained from an *S. typhimurium fim fliC fljB dam*_+803_::Mu*d*-Cam mutant. (E) The Coomassie-stained gel shows standard proteins (MW, left lane) and a fimbrial preparation (right lane). The molecular mass of relevant standard proteins is indicated on the left. The arrow indicates a single major protein band present in the fimbrial preparation. (F) The Western blot shows the fimbrial preparation (left lane) and purified His–StdA fusion protein (right lane). Note that due to its 6xHis tag, the His–StdA protein has a slightly greater molecular mass than native StdA (arrow). The molecular mass of standard proteins is indicated on the left.

Mutants that strongly expressed StdA were further analysed by electron microscopy to determine whether they elaborated fimbriae on their surface. When bacteria were cultured statically at 37°C in LB broth no fimbrial filaments were detected on the surface of the parental *S. typhimurium fim* mutant (AJB4) (data not shown), which is consistent with previous observations that type 1 fimbriae, but not thin-curled fimbriae, are expressed under this growth condition (Grund and Weber, 1988). As expected, introduction of the *dam*, *setB* and *rosE* mutations into a Δ*stdAB* derivative of AJB4 (CD21) did not result in expression of StdA (data not shown). However, surface appendages were visualized by negative staining on the surface of the *dam* (CD2R), *setB* (CD15R) and *rosE* (CD8R) mutants identified by SIMPLE (Fig. 5A). The identity of these surface structures was further investigated by immunogold labelling with anti-StdA serum. Gold particles labelled filamentous surface structures on the surfaces of the *S. typhimurium dam*_+803_::Mu*d*-Cam (CD2R), *rosE*::Mu*d*-Cam (CD8R) and *setB*::T-POP (CD15R) mutants, demonstrating that StdA was incorporated into fimbrial structures in these strains (Fig. 5B–D). In contrast, labelling with rabbit anti-StdA serum and goat anti-rabbit gold conjugate did not result in deposition of gold particles on the surface of the parental *S. typhimurium fim* mutant (AJB4) (data not shown). Immunoelectron microscopy thus provided the first evidence that the *std* operon encodes fimbriae in *S. typhimurium*.

Fimbriae were purified from an *S. typhimurium fim fliC fljB dam*_+803_::Mu*d*-Cam mutant (to prevent contamination with flagella and type 1 fimbriae) after their removal from the surface by mechanical shearing. Separation of proteins by SDS-PAGE followed by Coomassie blue staining revealed that the fimbrial preparation contained a single protein band with an apparent molecular mass that was similar to that predicted for the mature StdA protein (16 kDa) (Fig. 5E). In addition to a fimbrial usher (StdB) and a chaperone (StdC), the *std* operon encodes another putative subunit (StdD) whose predicted molecular mass was considerably larger than that of StdA. Western blot analysis showed that the protein band present in the fimbrial extract reacted with anti-StdA antiserum (Fig. 5F). Collectively, these data suggest that StdA is the major subunit of fimbriae encoded by the *std* operon.

**Fig. 6:**
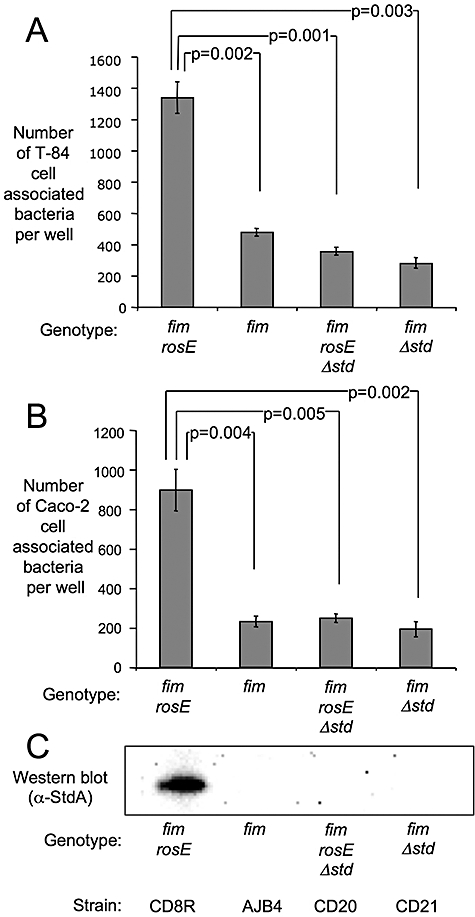
Adherence of *S. typhimurium* strains to human colonic epithelial cells. A and B. Adherence to T84-cells (A) or CaCo-2 cells (B) was determined at 4°C to prevent bacterial invasion. Data are shown as averages of cell-associated bacteria ± standard deviation. Statistical significance of differences is indicated by brackets. C. Expression of StdA in the bacterial inoculum was investigated by Western blot with anti-StdA serum.

To investigate the function of fimbriae encoded by the *std* operon, we studied bacterial adhesion to two human colonic epithelial cell lines, T-84 cells and CaCo-2 cells. Compared with its parent (AJB4), the *rosE*::Mu*d*-Cam mutant (CD8R) was recovered in significantly higher numbers from both CaCo-2 cells and T84 cells (Fig. 6A and B). This increased recovery was due to the presence of *std*, because deletion of this fimbrial operon abolished adherence of the *rosE*::Mu*d*-Cam mutant (CD20). Western blot analysis of the bacterial inoculum confirmed that only adherent bacterial cultures expressed StdA (Fig. 6C). These data demonstrated that fimbriae encoded by the *std* operon mediate attachment to human colonic epithelial cells.

### RosE is a negative regulator of StdA expression

**Fig. 7:**
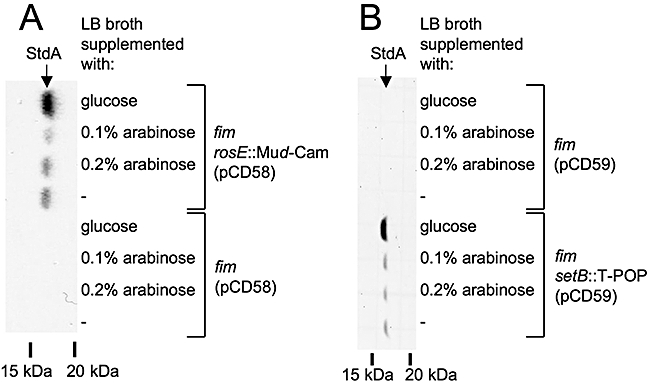
Complementation of *S. typhimurium* mutants expressing StdA. A. Complementation of the CD8R mutant (*fim rosE*::Mu*d*-Cam) (top) and its parent AJB4 (*fim*) (bottom) with the *rosE* gene cloned under control of the arabinose promoter (pCD58). A Western blot detected with anti-StdA serum is shown on the left. The molecular mass of standard proteins is indicated at the bottom. The presence or absence of glucose or arabinose is indicated on the right. B. Complementation of the CD15R mutant (*fim setB*::T-POP) (bottom) and its parent AJB4 (*fim*) (top) with the *setB* gene cloned under control of the arabinose promoter (pCD59). A Western blot detected with anti-StdA serum is shown on the left. The molecular mass of standard proteins is indicated at the bottom. The presence or absence of glucose or arabinose is indicated on the right.

Complementation of *dam* mutations was not attempted because both gene inactivation and introduction of the cloned gene can result in the same phenotype (e.g. increased spontaneous mutation rates in *S. typhimurium*) (Torreblanca and Casadesus, 1996). To complement the *rosE*::Mu*d*-Cam and *setB*::T-POP mutations, both open reading frames were PCR amplified along with their ribosome binding sites, but without their promoter sequences, and cloned in vector pBAD30 behind the *E. coli* arabinose promoter (P_BAD_). The resulting plasmids (pCD58 and pCD59 respectively) were introduced into the respective *S. typhimurium* mutants and strains were cultured in the presence of glucose (to repress expression from P_BAD_) or in the presence of arabinose (to induce expression from P_BAD_). Complementation of the *setB*::T-POP mutant (CD15R) with the cloned *setB* gene (pCD59) resulted in reduced StdA expression detected by Western blot. Similarly, introduction of the cloned *rosE* gene (pCD58) into the *rosE*::Mu*d*-Cam (CD8R) mutant resulted in repression of StdA expression (Fig. 7). These data suggested that inactivation of *rosE* and *setB* were responsible for increased expression of StdA strains CD8R and CD15R respectively.

**Fig. 8:**
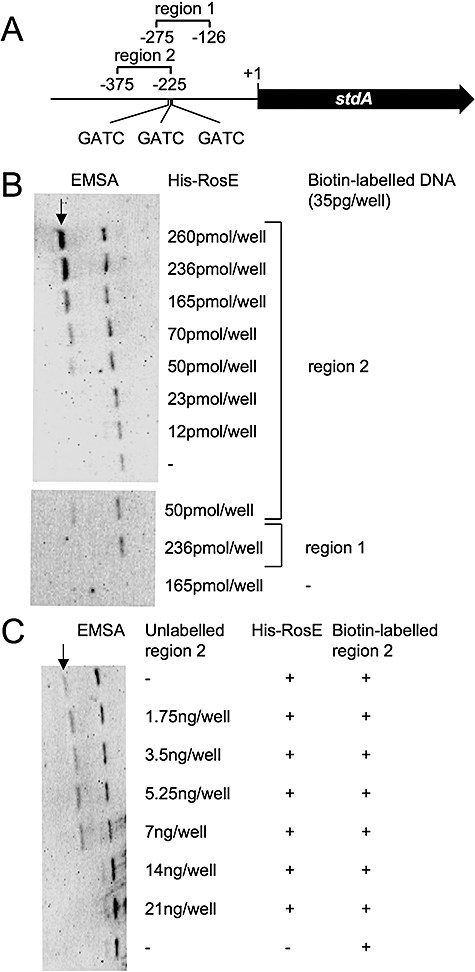
Electrophoretic mobility shift assay (EMSA) of DNA regions located upstream of the *stdA* start codon using purified His–RosE protein. A. The locations of DNA fragments (brackets) used for the EMSA (region 1 and region 2) are shown relative to the *stdA* open reading frame (filled arrow). The positions of three Dam methylation sites (GATC) present in the putative *std* promoter region are indicated. B. EMSA of biotin-labelled DNA (region 1 or region 2) and different concentrations of His–RosE. Shifted DNA fragments are indicated by an arrow. C. Competitive EMSA of biotin-labelled DNA (region 2, 35 pg well^−1^) in the presence (+) or absence (−) of purified His–RosE protein (70 pmol well^−1^) and the indicated amounts of unlabelled competitor DNA (region 2). Shifted DNA fragments are indicated by an arrow.

As RosE showed homology to transcriptional regulators, we investigated whether this protein would bind to a DNA region upstream of the *stdA* open reading frame. To this end, a fusion protein between RosE and a histidine tag (His–RosE) was constructed and purified. Purified His–RosE protein was tested for its ability to bind the *stdA* upstream DNA region using an electrophoretic mobility shift assay (EMSA). Addition of increasing concentrations of His–RosE to a biotin-labelled PCR product containing nucleotides −225 to −375 relative to the *stdA* start codon (Fig. 8A, region 2) resulted in appearance of a band with higher molecular weight (Fig. 8B). In contrast, His–RosE did not result in an electrophoretic mobility shift of a biotin-labelled DNA region comprising nucleotides −275 to −126 relative to the *stdA* start codon (Fig. 8A, region 1). The electrophoretic mobility shift of biotin-labelled DNA region 2 could be inhibited by pre-incubation of His–RosE with unlabelled DNA region 2, indicating specific binding of His–RosE to the *stdA* upstream DNA region (Fig. 8C).

Collectively, our data showed that RosE bound to a DNA region upstream of the *stdA* start codon (Fig. 8), repressed transcription from the *stdA* promoter (Fig. 4) and prevented expression (Fig. 7) and assembly of StdA into fimbrial filaments (Fig. 5). These results identified RosE as a transcriptional regulator of Std fimbrial expression in *S. typhimurium*.

### RosE-mediated repression of the *std* operon is required for full infectivity of *S. typhimurium*

**Fig. 9:**
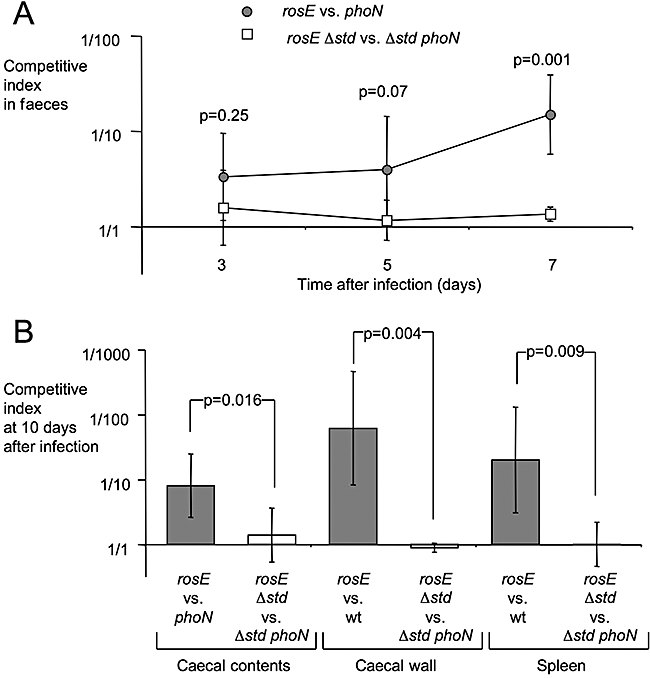
Effect of a mutation in *rosE* on the ability of *S. typhimurium* to colonize genetically resistant (CBA/J) mice. A. Mice were infected with a 1:1 mixture of the *S. typhimurium phoN* mutant and a *rosE* mutant (grey circles) or with a 1:1 mixture of *std phoN* mutant and a *std rosE* mutant (open squares) and bacteria were recovered from faeces over time. Data are shown as geometric means of competitive indices ± standard deviation. Statistical significance of differences between both competitive infection experiments are indicated by *P*-values. B. Mice were infected with a 1:1 mixture of the *S. typhimurium phoN* mutant and a *rosE* mutant (grey bars) or with a 1:1 mixture of *std phoN* mutant and a *std rosE* mutant (open bars) and bacteria were recovered from organs at 10 days after infection. Data are shown as geometric means of competitive indices ± standard deviation. Statistical significance of differences between both competitive infection experiments are indicated by *P*-values.

Next we wanted to determine whether constitutive expression of the *std* operon in a *rosE* mutant would alter the course of infection in mice. To this end, the *rosE*::Mu*d*-Cam insertion was introduced into the *S. typhimurium* wild type (SR11) to give rise to strain CD30. The *S. typhimurium* wild type was marked with a mutation in *phoN* (CD31), encoding alkaline phosphatase. Inactivation of *phoN* abolishes the ability to cleave 5-bromo-4-chloro-3-indolyl phosphate (XP). Growth on LB agar plates supplemented with XP thus provided an easy means to distinguish between mutants carrying a mutation in *rosE* (PhoN^+^ blue colonies) and CD31 (PhoN^-^ white colonies). Inactivation of *phoN* does not reduce the ability of *S. typhimurium* to colonize organs or faeces of mice during competitive infections (Kingsley *et al*., 2003; Weening *et al*., 2005). *A* group of six genetically resistant mice (CBA/J) was inoculated with a 1:1 mixture of the *S. typhimurium phoN* mutant (CD31) and a *rosE* mutant (CD30). Recovery of bacteria from the faeces showed that the *rosE* mutant was recovered at significantly reduced numbers at 7 days after infection (Fig. 9A). Determination of the bacterial tissue load at 10 days after infection revealed a significant competitive disadvantage of the *rosE* mutant for colonizing caecal contents, caecal wall and the spleen (Fig. 9B).

We next determined whether reduced recovery of the *rosE* mutant from the faeces and organs of mice was due to uncontrolled expression of the *std* operon or due to altered expression of other genes that may be regulated by RosE. We reasoned that deletion of the *std* fimbrial biosynthesis genes from both competing strains would eliminate a competitive defect only if recovery of the *rosE* mutant was due to uncontrolled expression of the *std* operon. The *std* operon was deleted from the *S. typhimurium phoN* mutant (CD31) and the *rosE* mutant (CD30) to give rise to strains CD32 and CD33 respectively. A group of six genetically resistant mice (CBA/J) was inoculated with a 1:1 mixture of the *std phoN* mutant (CD32) and the *std rosE* mutant (CD33). Recovery of bacteria from the faeces and organs of mice showed that the *std rosE* mutant (CD33) was fully able to compete with the *std phoN* mutant (CD32) for colonization of mice (Fig. 9). These data provided evidence that RosE-mediated repression of the *std* operon is required for full virulence of *S. typhimurium* in mice.

## Discussion

Sequence analysis has identified multiple putative fimbrial operons in the *S. typhimurium* genome that remain poorly characterized (Emmerth *et al*., 1999; Folkesson *et al*., 1999; Morrow *et al*., 1999; Tsolis *et al*., 1999; McClelland *et al*., 2001). We have recently developed a new method, termed SIMPLE, that can be used to isolate fimbriated *S. typhimurium* mutants from a random transposon library (Nuccio *et al*., 2007). Here we have applied this method for the first time to a putative fimbrial operon, *stdABCD*, which was identified in the *S. typhimurium* genome by whole genome sequencing. The SIMPLE method proved to be a powerful approach for isolating mutants expressing fimbriae encoded by the *std* operon *in vitro*. The isolation of such mutants represents an important first step in the characterization of fimbriae encoded by the *std* operon and opened the way for functional studies on their binding specificity. Specifically, *in vitro* expression of the *std* operon enabled us to demonstrate that the encoded fimbriae mediate attachment of *S. typhimurium* to human colonic epithelial cell lines (i.e. T-84 and CaCo-2).

While this work was in progress, Balbontin *et al*. (2006) demonstrated that transcription of the *std* operon is repressed by Dam methylation in *S. typhimurium*. Our data confirmed and extended this observation by showing that mutations in *dam* resulted in expression of StdA filaments on the bacterial cell surface. The Dam protein methylates adenine residues located within the palindromic sequence GATC (Razin and Friedman, 1981). Inspection of the *std* promoter region reveals three GATC sites arranged in the motif 5′-acGATCa-(N_6_)-tcGATCgt-atcGATCta-3′ located upstream of a putative *stdA* promoter (Balbontin *et al*., 2006) (211 bp upstream of the *stdA* start codon). A similar arrangement of GATC sites and flanking regions is found in the promoter region of the *E. coli agn43* gene, which contains the sequence 5′-acGATCa-(N_12_)-tgGATCgt-(N_4_)-atcGATCga-3′ located downstream of the transcriptional start site (32 bp upstream of the *agn43* start codon) (Waldron *et al*., 2002; Wallecha *et al*., 2002). Dam-mediated methylation of these GATC sites is part of a phase variation mechanism controlling expression of antigen 43, an adhesin of the autotransporter family (Henderson and Owen, 1999; Haagmans and van der Woude, 2000). However, while *E. coli dam* mutants do not express antigen 43, *S. typhimurium dam* mutants did express StdA, which illustrated that regulation of Std fimbriae differed from that of the *E. coli* autotransporter.

A novel transcriptional regulator of *std* expression identified in this study is RosE, a protein with homology to the arginine repressor ArgR of *E. coli* (Lim *et al*., 1987). Analysis of a P_*rosE*_::*lacZ* reporter construct and complementation of a *rosE*-40::Mu*d*-Cam mutant (CD8R) with the cloned *rosE* gene suggested that RosE repressed transcription of the *std* operon. Furthermore, EMSAs showed that RosE bound a DNA region located between base pairs −375 and −225 relative to the *stdA* start codon. For comparison, the GATC sites implicated in Dam-mediated repression of *stdA* expression (Balbontin *et al*., 2006) are located between base pairs −239 and −214 relative to the *stdA* start codon. Collectively, these data suggested that similar to its homologue ArgR, the RosE protein functioned as a transcriptional repressor. The *rosE* gene is located on a genetic islet that is well conserved among the genus *Salmonella*, thus making it likely that co-ordination of gene expression by RosE is a general feature of *Salmonella* serotypes.

While our data show that RosE prevented StdA expression *in vitro*, characterization of an *S. typhimurium*Δ*stdAB* mutant in the mouse model suggests that RosE-mediated suppression must be relieved under some conditions *in vivo*. The *S. typhimurium*Δ*stdAB* mutant has a competitive defect in colonizing the caecum of mice and in being shed with the faeces (Weening *et al*., 2005), suggesting that fimbriae encoded by the *std* operon are expressed in the intestinal lumen. StdA seroconversion of mice infected with *S. typhimurium* provides further indirect evidence for *in vivo* expression of fimbriae encoded by the *std* operon (Humphries *et al*., 2005). Interestingly, we found that a mutation in *rosE* resulted in a competitive defect of *S. typhimurium* during colonization of mice. This competitive colonization defect was no longer observed when *std* biosynthesis genes were deleted from both competing strains. These data suggest that a mutation in *rosE* resulted in attenuated mouse virulence of *S. typhimurium* because fimbriae encoded by the *std* operon were expressed *in vivo* at an inappropriate time or location. Deletion of the *std* operon results in a competitive defect of *S. typhimurium* in colonizing the caecum of mice, while colonization of the spleen is not altered (Weening *et al*., 2005). In contrast, the uncontrolled expression of the *std* operon in a *rosE* mutant reduced the ability of *S. typhimurium* to colonize both intestinal sites (i.e. the caecum) and extra-intestinal sites (i.e. the spleen). Collectively, these data illustrate that a tight control of *std* fimbrial expression is critical during host pathogen interaction *in vivo.*

## Experimental procedures

### Bacterial strains, media and growth conditions

*Salmonella enterica* serotype Typhimurium strains used in this study are shown in Table 1. An *S. typhimurium fim fliC fljB dam*_+803_::Mu*d*-Cam mutant was generated by transducing the *fliC* and *fljB* mutations of *S. typhimurium* strain EHW26 (Raffatellu *et al*., 2005) into strain CD2R. *E. coli* strains DH5α MCR (Gibco BRL) and TOP10 (invitrogen) were used for cloning experiments. Insertions in the *phoN* gene were introduced into *S. typhimurium* strains by P22-mediated transduction from strain AJB715.

**Table 1:** *S. typhimurium* strains used in this study.

Strain	Genotype	Source or reference
SR11	Xylose fermenting mutant of wild-type isolate BA2	Schneider and Zinder (1956)
ADH17	SR11, Δ*stdAB*::Km	Humphries *et al*. (2003)
CD30	SR11, *rosE*::Mu*d*-Cam	This study
CD31	SR11, *phoN*::Km	This study
CD32	SR11, *phoN*::Km Δ*stdAB*::Km	This study
CD33	SR11, *rosE*::Mu*d*-Cam Δ*stdAB*::Km	This study
AJB4	SR11, nalidixic acid resistant, *fim*	Bäumler *et al*. (1996)
CD6R	AJB4, *csgC*::T-pop	This study
CD7R	AJB4, *yfbQ*::T-pop	This study
CD8R	AJB4, *rosE*::Mu*d*-Cam	This study
CD10R	AJB4, *dam*_−40_*::*Mu*d*-Cam	This study
CD12R	AJB4, *damX::*Mu*d*-Cam	This study
CD15R	AJB4, *setB*::T-pop	This study
DB3R	AJB4, *dam*_+36_*::*Mu*d*-Cam	This study
CD20	AJB4, *rosE*::Mu*d*-Cam Δ*stdAB*::Km	This study
CD21	AJB4, Δ*stdAB*::Km	This study
CD2R	AJB4, *dam*_+803_*::*Mu*d*-Cam	This study
CD3	CD2R, *fliC fljB*	This study
ATCC14028	Wild-type isolate from cattle	American Type Culture Collection
IR715	Nalidixic acid-resistant derivative of ATCC14028	Stojiljkovic *et al*. (1995)
EHW26	IR715, *fliC*::Tn*10 fljB5001*::Mu*d*J	Raffatellu *et al*. (2005)
AJB715	IR715, *phoN*::Km	Kingsley *et al*. (2003)
LT2	ATCC 700720	Lilleengen (1948)
TE3461	LT2, *hisD9953*::Mu*d*-Cam *hisA9944*::Mu*d*-1	Elliott (1993)
TH3923	LT2, pJS28(Carb^R^, P22-9+)/F′114ts Lac+*zzf-20*::Tn*10*[*tetA*::MudP](Tc^S^) *zzf-3823*::Tn*10*dTc[del-25](T-POP)/*leuA414 hsdSB* Fels2−	Rappleye and Roth (1997)

All strains were cultured aerobically or statically at 37°C in LB broth (10 g l^−1^ tryptone, 5 g l^−1^ yeast extract, 5 g l^−1^ NaCl). For the SIMPLE approach, bacteria were cultured in LB buffered to pH 7 with 100 mM 2-(N-morpholino)ethanesulphonic acid (MES) (Nicholson and Low, 2000). When appropriate, antibiotics were added at the following concentrations (mg l^−1^): kanamycin (Km), 100; nalidixic acid (Nal), 50; carbenicillin (Carb), 100; tetracycline (Tc), 20. To detect expression of beta-galactosidase on agar plates, 5-bromo-4-chloro-3-indolyl-β-d-galactopyranoside (X-Gal) was added at a concentration of 40 mg l^−1^. For detection of alkaline phosphatase (PhoN) activity in *S. typhimurium* 30 mg l^−1^ XP was added to LB agar plates.

### Genetic techniques

Generalized transducing phages P22 HT *int-105* and KB1 *int* were used to generate lysates of *S. typhimurium* as previously described (Miller, 1972). Transductants were streaked for single colonies on Evans blue Uridine (EBU) agar (Bochner, 1984) and phage-free colonies were cross-streaked against P22 H5 (for P22 HT *int-105*), or KB1 *int* (for KB1) to confirm phage sensitivity.

Mu*d*-Cam insertion mutants of *S. typhimurium* strain AJB4 were isolated using the method of Hughes and Roth (1988). In brief, a P22 HT *int-105* lysate of TE3461 (Elliott, 1993) was used to transduce *S. typhimurium* to chloramphenicol resistance. Mu*d*-Cam insertions were selected on LB+Cm plates (approximately 1000–2000 mutants per plate) and pooled by flooding with 5 ml of LB broth and re-suspending colonies.

Tn*10*dTc[del-25](T-POP) insertion mutants of *S. typhimurium* AJB4 were generated as described previously (Rappleye and Roth, 1997), using a P22 lysate grown on TH3923 to transduce into *S. typhimurium* AJB4 carrying plasmid pNK2880 (Rappleye and Roth, 1997; Lee *et al*., 2007). Transductants were selected on LB+Tc agar (approximately 1000–2000 mutants per plate) and pooled by flooding with 5 ml of LB broth and re-suspending colonies.

### SIMPLE protocol

SIMPLE was performed as described previously (Nuccio *et al*., 2007). In brief, 100 μl aliquots of BioMag Protein G particles (Qiagen) were washed three times in 750 μl of TN buffer (0.1 M Tris-HCl, 0.15 M NaCl, pH 7.5) and incubated for 1 h at room temperature in 100 μl of pre-absorbed anti-StdA serum on an automatic roller. The particles were washed as described above and incubated at room temperature for half an hour with constant inversion on an automatic roller in 650 μl of Particle-Blocking (PB) buffer (TN buffer + 1% casein, prepared fresh). Bacteria (500 μl of a culture grown statically at 37°C for 2 days in LB-MES pH 7) were added and the tubes were incubated for 1 h at room temperature with constant inversion. The particles were washed three times with PB buffer by gentle inversion and re-suspended in 1 ml of PBS (to generate serial 10-fold dilutions that were spread on agar plates) or in the appropriate growth medium (to incubate bacteria statically at 37°C for 2 days and use the resulting culture to repeat the above protocol). For each mutant pool, 10 single colonies from a plate were analysed by Western blot using anti-StdA serum.

### Western blotting

The polyclonal rabbit anti-StdA serum (Humphries *et al*., 2003) was diluted 1:5 in PBS supplemented with 0.2% sodium azide and pre-absorbed with ADH17 as described previously (Gruber and Zingales, 1995). For Western blot analysis, 10 μl containing approximately 2 × 10^8^ colony-forming units (cfu) re-suspended in PBS was mixed with an equal volume of sodium dodecyl sulphate (SDS)-polyacrylamide gel electrophoresis (PAGE) loading buffer and boiled for 10 min. These whole-cell lysates were separated by 15% SDS-PAGE, transferred to Immobilon-P (Millipore) membranes using a Trans-Blot semi-dry transfer cell (Bio-Rad) and incubated with anti-StdA serum diluted 1:500 (final). Binding of anti-StdA serum was detected with Goat anti-Rabbit-Alkaline Phosphatase conjugate and the Immun-Star chemiluminescent substrate (Bio-Rad). Bands were visualized with a BioSpectrumAC Imaging System (UVP).

### Cloning of transposon flanking DNA

DNA regions adjacent to the transposon insertion sites were amplified by an inverse PCR as described previously (Bäumler *et al*., 1994). Genomic DNA was digested with either AluI or TaqI and ligated with T4 DNA Ligase. DNA flanking Mu*d*-Cam insertions was amplified using primer 5′-CCGAATAATCCAATGTCCTCCCGGT-3′ in combination with either 5′-AGTGCGCAATAACTTGCTCTCGTTC-3′ (for TaqI-digested DNA) or 5′-CGAAAAACAAAAACACTGCAAATCATTTCAATAAC-3′ (for AluI-digested DNA). DNA flanking T-POP insertions was amplified using primers 5′-CGCTTTTCCCGAGATCATATG-3′ and TPOP-AluTaq, 5′-GCACTTGTCTCCTGTTTACTCC-3′ (for AluI-digested DNA) and PCR SuperMix HiFi (Invitrogen). PCR products were gel-purified (QIAEX II gel purification kit, Qiagen) and cloned into pCR2.1 with the TOPO TA cloning kit (Invitrogen). The inserts of the resulting plasmids were sequenced by SeqWright (Houston, TX).

### Complementation

The *rosE* gene was PCR amplified with the primers 5′-GAGCTCTAAGGTGCATTTATGAAGGA-3′ and 5′-AAGCTTACTCATCGCAAACGGTTCTTA-3′, cloned into pCR2.1 and the insert excised with SacI and HindIII and cloned into vector pBAD30 (Guzman *et al*., 1995) to give rise to plasmid pCD58. The *setB* gene was PCR amplified with the primers 5′-GAATTCCGTAAACTCCGCCTCTCTTCACAC-3′ and 5′-AAGCTTGCTGAAATGTGTCGAAGAGTAAA-3′, cloned into pCR2.1 and the insert excised with EcoRI and HindIII and cloned into vector pBAD30 to give rise to plasmid pCD59. The plasmids were introduced into *S. typhimurium* strains by electroporation. The resulting strains were grown overnight at 37°C in LB+Carb supplemented with 10 mM glucose. This overnight culture was used to inoculate (1:100) LB+Carb or LB+Carb supplemented with 0.2% of glucose, 0.1% arabinose or 0.2% arabinose. Cultures were grown at 37°C statically to an OD_600_ of 0.4–0.6 and StdA expression assessed by Western blot with rabbit anti-StdA serum (Humphries *et al*., 2003).

### Electron microscopy

For microscopy, bacteria were grown for 2 days in a static culture, washed twice in PBS and re-suspended in EM grade water (EM Science) at a titre of approximately 1 × 10^9^ cfu ml^−1^. Bacteria were allowed to attach to a formvar/carbon-coated grid (EM Science) for 2 min. For negative staining of fimbriae, the grids were incubated for 1 min in 1% uranyl-acetate (UA). For immunogold labelling the grids were incubated for 20 min with rabbit anti-StdA serum diluted 1/250 in PBS containing 1% bovine serum albumin (BSA). Grids were washed five times for 1 min in PBS containing 1% BSA. Grids were then incubated for 20 min in goat anti-rabbit 10 nm gold conjugate (EM Science) diluted 1/20 in PBS containing 1% BSA. Grids were washed three times for 1 min in PBS containing 1% BSA and three times for 1 min in EM grade water. Grids were incubated for 1 min with 1% UA before they were analysed by electron microscopy.

### Purification of fimbriae

An *S. typhimurium fim fliC fljB dam*_+803_::Mu*d*-Cam mutant was grown statically in 2 l of LB broth at 37°C overnight, harvested by centrifugation and re-suspended in 10 ml of 0.5 mM Tris 75 mM NaCl. StdA fimbriae were separated from the cells by mechanical shearing in a blender for three 1 min periods, after which cells and cellular debris were removed by centrifugation (3500 r.p.m. 30 min, 4°C). The supernatant was collected and passed through a 0.45 μm filter (Millipore), and (NH_4_)_2_SO_4_ (60% final concentration) was added to precipitate the fimbriae. Precipitated fimbriae were recovered by centrifugation (14 000 r.p.m. 30 min, 4°C). The pellet was re-suspended in 50 μl of sterile water and was analysed by SDS-PAGE and Western blot.

### Tissue culture experiments

The colorectal carcinoma cell lines T84 (ATCC CCL-248) and Caco-2 (HTB-37) were obtained from the American Type Culture Collection. T84 cells were routinely cultured in Dulbecco's modified Eagle's medium (DMEM)-F12 medium (Gibco), containing 1.2 g l^−1^ sodium bicarbonate, 2.5 mM l-glutamine, 15 mM HEPES and 0.5 mM sodium pyruvate (Gibco), supplemented with 10% fetal calf serum (FCS). Caco-2 cells were maintained in Minimum essential medium (Eagle) (Gibco) containing 2.5 mM l-glutamine, 0.1 mM non-essential amino acids, 1 mM sodium pyruvate, Earle's balanced salt solution (BSS) and 15% FCS. For assays, T84 cells were seeded in 24-well plates at a density of approximately 1 × 10^5^ cells per well or in six-well plates at a density of 5 × 10^5^ cells per well and incubated for 48 h. Caco-2 cells were grown to confluency in 24-well plates for 5 days. Prior to adherence assays, epithelial cells were washed with ice-cold PBS. Ice-cold medium was added, cells were infected with bacteria (10^5^ cfu well^−1^) and allow to adhere for 1 h at 4°C (to prevent invasion). Non-adherent bacteria were removed by five washes with PBS and adherent bacteria re-suspended in PBS containing 1% (v/v) Triton X-100. Serial 10-fold dilutions were spread on LB agar plates to determine the number of cell-associated bacteria per well.

### β-Galactosidase assay

The promoter region of StdA was PCR amplified with the primers 5′-TCTAGACCTGAACTTTCCATCGAA-3′ and 5′-CCCGGGATATCCCCCAGCCTGCTG-3′. The PCR product was cloned into pCR2.1 (Invitrogen), the insert excised with XbaI and EcoRI and cloned into vector pUJ10 (deLorenzo *et al*., 1990) to give rise to plasmid pCD57. Plasmid pCD57 was introduced into *S. typhimurium* strains by electroporation. For β-galactosidase measurements, LB+Carb was inoculated 1:100 from an overnight culture and bacteria were grown to an optical density at 600 nm (OD_600_) of 0.4–0.6. The enzymatic activity of β-galactosidase in each culture was determined using standard methodology (Miller, 1972).

### Purification of His–RosE

An N-terminal 6xHis–RosE fusion protein was constructed using the cloning vector pQE30 (Qiagen). The *rosE* gene was PCR amplified using the primers 5′-GGATCCATGAAGGAATACGATGATTAC-3′ and 5′-GAGCTCTTATGAATTTAAATTCATTTTAAG-3′ and the product was cloned into pCR2.1 (Invitrogen). The insert was then cloned into the BamHI and SacI sites of pQE30 to give rise to plasmid pCD62. Plasmid pCD62 was electroporated into *E. coli* TOP10 and the resulting strain was grown overnight at 37°C in LB media supplemented with 10 mM glucose and carbenicillin (100 mg l^−1^) to maintain the plasmid. To induce the production of His–RosE, cells were grown to mid-log phase before IPTG was added to a final concentration of 2 mM. Cells were harvested by centrifugation after a 4 h incubation. His–RosE was purified by nickel-affinity chromatography (Ni-NTA, Invitrogen) according to the manufacturer's protocol, but after cell fragments had been removed from cell lysates by centrifugation, the supernatant was centrifuged at 15000 r.p.m. for 30 min to remove denatured protein before being applied to the affinity column. The fractions containing His–RosE were immediately aliquotted and frozen at −80°C in 5% sucrose. The His–RosE concentration was determined by measuring the absorbance at 280 nm.

### Electrophoretic mobility shift assay

Fragments of the region upstream of the *stdA* open reading frame were generated by PCR amplification using the primer sets 5′-CCTGAACTTTCCATCGAAAAA-3′, 5′-TAAAAAGTATTTCTTTGAT-3′ (region 1) and 5′-TCGATGATTATGATCGTAAT-3′, 5′-ATGGAAAAGCAAAACATA-3′ (region 2) which were biotinylated at the 5′ end (MWG Bioscences, High Point, NC). The PCR products were gel-purified in a vertical acrylamide gel. The vertical acrylamide gel was prepared by adding 0.1 ml of 50× Tris-acetate EDTA (242 g of Tris, 57.1 ml of glacial acetic acid, 37.2 g of Na_2_EDTA·2H_2_O in 1 l of distilled H_2_O), 0.66 ml of acrylamide/bis-acrylamide (37.5:1, Roche), 6 μl of TEMED and 30 μl of ammonium persulphate to 5.33 ml of distilled H_2_O. After electrophoresis, fragments were excised and eluted by electrophoresis in dialysis tubes for 15 min at 100 V. The DNA concentration was determined by spectrophotometry. The binding assays were performed in a total volume of 20 μl containing the following: 1 μg ml^−1^ Poly(dI-dC), 50% glycerol, 1% non-ylphenyl-polyethylene glycol (NP-40), 1.05 M KCl, 10 mM TRIS (pH 7.5), 1 mM DTT, 100 mM MgCl_2_, 200 mM EDTA, 35 pg of biotinylated DNA, various concentrations of His–RosE and unlabelled competitor DNA as required. Competitor DNA was generated by PCR amplification using the primers listed above without the biotin label. Binding reactions were allowed to incubate for 20 min at room temperature prior to adding loading buffer and separating fragments on a pre-run 5% acrylamide gel in Tris-borate EDTA buffer (Bio-Rad Precast Gels). Gels were run at 4°C at 100 V until the bromophenol blue dye front had migrated approximately three-fourths down the length of the gel. The DNA–protein complexes were transferred to a nylon membrane (Roche Applied Science) using a semi-dry blotter (Bio-Rad) at 15 V for 30 min. The DNA was UV cross-linked to the membrane, and biotinylated DNA on the membrane was detected using the LighShiftTM Chemiluminescent EMSA Kit (Pierce Biotechnology, Rockford, IL) according to the manufacturer's protocol.

### Animal experiments

Eight- to 12-week-old CBA/J (Jackson Laboratory) mice were used throughout this study. Bacteria were routinely cultured as standing overnight cultures prior to infection. In all experiments the bacterial titre of the inoculum was determined by spreading serial 10-fold dilutions on agar plates containing appropriate antibiotics and determining cfu. For competitive infection experiments, groups of five mice were infected by oral gavage with a 1:1 mixture of mutant and isogenic parent at a dose of approximately 5 × 10^8^ cfu per mouse. Faecal pellets were homogenized in 1 ml of PBS. The limit of detection was approximately 0.08 cfu per milligram of faeces. Caecum, caecal contents and spleen were harvested and homogenized in 5 ml of PBS pH 7.4. Dilutions of faecal pellets and homogenized organs were spread on LB agar plates containing the appropriate antibiotics. Agar plates were supplemented with XP to distinguished between colonies expressing PhoN and colonies that were PhoN negative. Data were normalized and competitive indices calculated by dividing the output ratio (cfu mutant/cfu wild type) by the input ratio (cfu mutant/cfu wild type). Competitive indices were converted logarithmically prior to the calculation of averages and statistical analysis. A Student's *t*-test was used to determine whether differences were statistically significant.
